# Osteocalcin as a predictor of bone fracture in children with chronic kidney diseases

**DOI:** 10.1007/s40620-025-02385-4

**Published:** 2025-09-03

**Authors:** Happy Sawires, Shrouk Abdallah, Mohamed Ramadan, Radwa Abdel-Halim, Yasmin Ramadan

**Affiliations:** 1 Pediatrics Department, Cairo University, Cairo, Egypt; 2 Ministry of Health, Cairo, Egypt; 3 Clinical & Chemical Pathology Department, Cairo University, Cairo, Egypt

**Keywords:** Osteocalcin, Bone alkaline phosphatase, CKD, Bone fracture

## Abstract

**Background:**

The associations between biochemical indicators of osteocalcin and bone health are fairly well established in adults through observational studies. In this context, our objective was to assess serum level of uncarboxylated osteocalcin (uOC) and its relation to the incidence of bone fractures other bone health indices in children with CKD.

**Methods:**

We enrolled 102 patients classified into two groups: group A: CKD without KRT; group B: CKD on regular HD. The patients were followed throughout the study period. The study’s endpoint was either the occurrence of a bone fracture or the conclusion of the study period. Another 22 healthy individuals were involved as a control group. We measured uOC and various indicators of bone health including bone-specific alkaline phosphatase (BAP) on the same day of the fracture or at the end of the study period.

**Results:**

uOC was found significantly higher in CKD children in comparison to the control group (*p* <0.001). Moreover, its level was significantly higher in the HD group compared to CKD without KRT group (*p* = 0.047). In patients with fractures, uOC and BAP were significantly higher compared with patients without fractures (*p* < 0.001 and 0.019, respectively). By logistic regression analysis, uOC was the only predictor of bone fractures (*p* = 0.027, OR = 1.011).

**Conclusion:**

Elevated uOC levels were observed in children with CKD who experienced fractures, and these levels showed a correlation with BAP. Furthermore, uOC appears to be a reliable indicator of bone fractures in this population.

**Graphical abstract:**

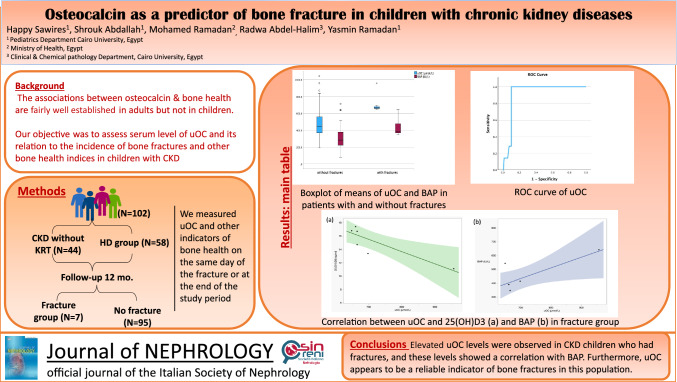

**Supplementary Information:**

The online version contains supplementary material available at 10.1007/s40620-025-02385-4.

## Introduction

Children with chronic kidney disease (CKD) often exhibit clinical signs of mineral bone disease (MBD), such as bone deformities and fractures, leading to prolonged disability. Many in this group experience abnormalities in skeletal mineralization, which can contribute to ongoing health issues [1].

Serum osteocalcin is a 5.6 kDa bone-derived protein with 49 amino acids and three gamma-carboxyglutamic residues. Its activation depends on vitamin K2-mediated carboxylation. The uncarboxylated form cannot bind hydroxyapatite, impacting bone mineralization and matrix integrity [2].

Patients with CKD often exhibit inadequate levels of vitamin K2 [3]. There are various factors contributing to this deficiency. Major iatrogenic causes of vitamin K2 deficiency in CKD patients include hemodialysis and the use of vitamin K2 antagonists [4]. Numerous studies have proposed that vitamin K2 may have a significant impact on the pathogenesis of severe complications in CKD patients, particularly concerning bone fractures and vascular calcification [5].

Evaluating bone health should be a crucial part of the clinical assessment of children with CKD. However, the tools currently available to clinicians have not been thoroughly researched and have significant limitations [6]. One of these biomarkers is bone-specific alkaline phosphatase, which is a homodimer that is anchored to the membrane of osteoblasts and matrix vesicles. When cleaved by a phospholipase, soluble (anchor-free) bone-specific alkaline phosphatase is released into the bloodstream and can serve as a biomarker for bone formation [7].

The associations between biochemical indicators of vitamin K status like osteocalcin and bone health have been well established in adults through observational studies. However, these relationships have not been as extensively studied or understood in children [8].

In this context, our objective was to assess serum level of uncarboxylated osteocalcin and its relation to the incidence of bone fractures and other bone health indices in children with CKD.

## Methods

This prospective cross-sectional study included 113 children of both genders and different ages with CKD. The patients were classified into 2 groups: group A; CKD without kidney replacement therapy (KRT) stages 3–5 and group B; CKD on regular hemodialysis (HD) for at least 3 months prior to the study start. The patients were convenience samples recruited from the pediatric nephrology department and the out-patient clinic at the Cairo University Children’s Hospital. The study also included 22 age- and gender-matched healthy children as a control group who were recruited from general pediatric out-patient clinics coming for routine check-ups. The study was approved by the local ethics committee and was conducted according to the Declaration of Helsinki. Written informed consent was obtained from the patients’ caregivers.

The CKD diagnostic criteria were based on the guidelines proposed by the Kidney Disease Outcomes Quality Initiative (K/DOQI) of the National Kidney Foundation, and subjects were classified according to estimated glomerular filtration rate (eGFR) [ml/min/1.73 m^2^] as follows: stage 3, eGFR = 30–59, stage 4, eGFR = 15–29 and stage 5, eGFR < 15. eGFR was assessed according to the Schwartz formula (plasma creatinine was measured by Jaffe’s method) [9].

None of the enrolled patients in this study received vitamin K supplementation, warfarin, calcimimetics, steroids, probiotics or bisphosphonates. Vitamin D derivatives, calcium supplements and phosphate binders were given according to their needs. Patients that had previous fractures, metabolic bone diseases, acute significant illness, active infection requiring antibiotic therapy within 2 weeks prior to the blood sampling or malignancy were excluded from the study.

The study tracked patients from February 2023 through the end of January 2024. The study endpoint was either the occurrence of a bone fracture—whether spontaneous or from minor trauma—or the conclusion of the study period. During follow-up, 6 patients were lost to follow-up, 4 patients developed infections that required antibiotic treatment within 2 weeks prior to sampling and one patient sustained a fracture due to an accident. The final cohort included 44 patients in group A and 58 patients in group B.

Demographic data were collected from the study cohort and the anthropometric measurements were plotted and calculated with the standard z-score using WHO Anthro software for WHO Growth Reference for children less than 5 years of age, while WHO AnthroPlus software was used for the global application of the WHO Growth Reference for 5–19 years [10]. All participants were evaluated with regard to their medical history and documentation of bone fractures, whether linked to minor injuries or occurring spontaneously.

### Biochemical analysis

Fasting blood samples were collected on the same day as the fracture (within 24 h of the fracture) or at the end of the study period (pre-dialysis for patients on HD) to measure serum calcium (Ca), phosphorus (P), blood urea nitrogen (BUN), and creatinine. Serum samples were allowed to clot at room temperature then centrifuged at 3000xg for 10 min to assay uncarboxylated osteocalcin, 25 (OH) D_3_, and bone-specific alkaline phosphatase by ELISA using Sun Red (Shanghai Sun Red Biological Technology Co., Ltd). Two ml of blood in a sterile ethylene diamine tetra acetate (EDTA) tube were collected and plasma was separated immediately so as to assay intact parathyroid hormone (PTH) by chemiluminescence assay using IMMULITE 1000 system (Simens Healthineers, Germany). Both serum uncarboxylated osteocalcin and bone-specific alkaline phosphatase were measured in all patients at the start of the study for comparison.

The primary outcome was to assess serum levels of uncarboxylated osteocalcin in children with CKD. The secondary outcomes of this study were to determine the correlations between serum levels of uncarboxylated osteocalcin and the incidence of bone fracture and other markers of bone health such as Ca, P, bone-specific alkaline phosphatase, PTH and 25 (OH)D_3_.

### Statistical analysis

Numerical data were presented as mean and standard deviation, while categorical data were presented as frequencies and percentages. The Independent T test and Mann–Whitney test were used to compare parametric and non-parametric variables, respectively. The Pearson correlation coefficient was used to determine significant correlations between quantitative data. Logistic regression analysis was performed to demonstrate the effects of different variables on the likelihood of bone fractures. Receiver operating characteristic (ROC) curve analysis was used to assess the accuracy, sensitivity and specificity of uncarboxylated osteocalcin. The significance level was set at *p* < 0.05. Statistical analysis was performed with SPSS 29.0 (statistical package for scientific studies) for Macintosh.

## Results

We enrolled 102 patients with a mean age of 6.37 ± 3.11 years, while mean age in the control group was 6.95 ± 2.51 years (*p* = 0.431). Bone fractures occurred in 7 patients (incidence 6.8%); one patient in group A and 6 patients in group B. All the patients had long bone fractures (3 with fibular fractures, 2 with tibial fractures and 2 with midshaft ulnar fractures). Descriptive characteristics and biochemical analysis data of the two patient groups are provided in Table 1.

**Table 1:** Descriptive characteristics and biochemical analysis data of the different groups

	Group A	Group B	*p* value
	(44 patients)	(58 patients)	
Age (years)	6.32 ± 2.41	7.12 ± 2.52	0.142
Gender			
Female (%)	14 (31.8%)	20 (34.5%)	0.834
Male (%)	30 (69.9%)	38 (65.5%)	
Original renal disease			
Obstructive uropathy	14 (31.8%)	17 (29.3%)	0.118
Chronic GN	10 (22.7%)	11 (19%)	
MAHA	7 (15.9%)	7 (12.1%)	
Nephronophthisis	4 (9.1%)	8 (13.8%)	
Others	9 (20.5%)	15 (25.9%)	
BMI z-score*	0.65 ± 2.63 (-6.53/ 7.04)	0.6 ± 2.12 (− 5.23 / 7.04)	0.927
BUN (mg/dl)	74.11 ± 27.23	138.53 ± 41.77	< 0.001
Creatinine (mg/dl)	1.7 ± 0.43	4.83 ± 1.83	< 0.001
eGFR (ml/min/1.73m^2^)	32.33 ± 10.28		
Calcium (mg/dl)	9.51 ± 0.86	9.24 ± 0.83	0.099
Phosphorus (mg/dl)	6.21 ± 1.03	6.77 ± 1.20	0.015
PTH^*^ (pg/ml)	152.38 ± 108.52 (65/390)	144.90 ± 73.76 (64/390)	0.680
uOC-base (µmol/L)	400.66 ± 106.88	447.38 ± 124.44	0.024
uOC (µmol/L)	427.55 ± 133.62	539.58 ± 183.88	0.002
25 (OH) D_3_ (ng/ml)	23.38 ± 4.37	18.47 ± 6.94	< 0.001
BAP-base (IU/L)	289.5 ± 116.13	332.67 ± 126.34	0.04
BAP (IU/L)	308.82 ± 131.74	334.78 ± 139.12	0.342

^*^Data presented with interquartile range, Mann–Whitney test was used for comparison

*BAP* bone alkaline phosphatase; *BMI* body mass index; *BUN* blood urea nitrogen; *eGFR* estimated glomerular filtration rate; *MAHA* microangiopathic hemolytic anemia; *PTH* parathormone; uOC uncarboxylated osteocalcin

Uncarboxylated osteocalcin was found to be significantly higher in all patients (491.26 ± 172.61) compared to the control group (313.35 ± 123.33) (*p* < 0.001). Moreover, levels were significantly higher in the HD group compared to CKD without KRT group (*p* = 0.047) (Supplementary Fig. 1).

**Fig. 1:**
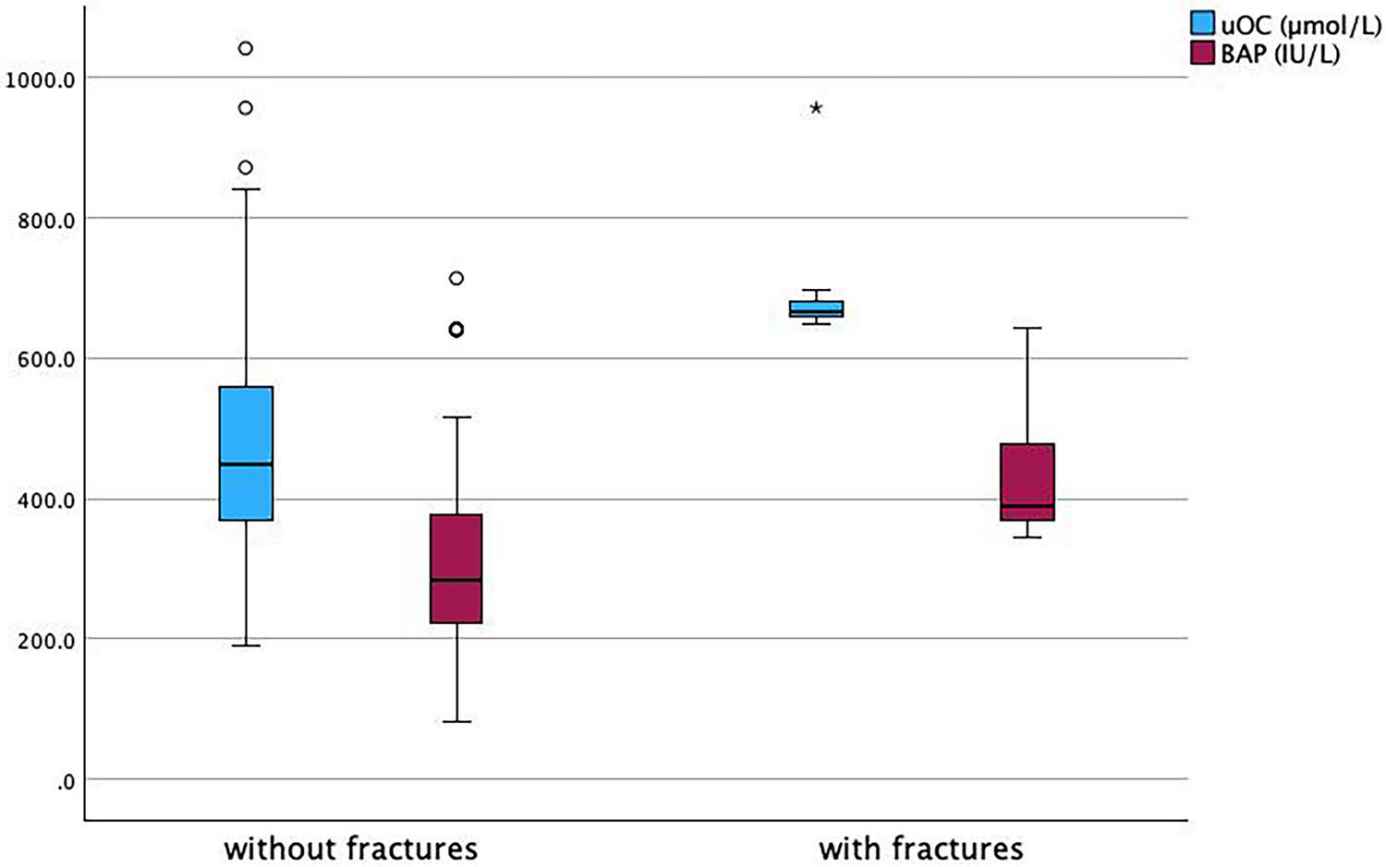
Boxplot of means of uncarboxylated carboxylation (uOC) and bone-specific alkaline phosphatase (BAP) in patients with and without fractures

It was observed that patients with fractures had statistically significant lower eGFR and 25(OH)D_3_ levels (*p* = 0.036 and 0.046, respectively), as well as higher levels of uncarboxylated osteocalcin and bone-specific alkaline phosphatase (*p* < 0.001 and 0.019, respectively). However, there was no statistically significant difference in age, BUN, creatinine, calcium, phosphorus, and PTH between patients with and without fractures (Table 2 and Fig. 1).

**Table 2:** Comparison between patients with and without fractures

	Patients with fractures (*N* = 7)	Patients without fractures (*N* = 95)	*p* value
Age (years)	6.29 ± 2.87	6.43 ± 3.26	0.917
BMI z-score*	− 0.42 ± 2.15 (− 2.62/ 3.87)	0.7 ± 2.38 (− 6.53 / 7.04)	0.229
BUN (mg/dl)	113.57 ± 27.06	110.53 ± 49.54	0.796
Creatinine (mg/dl)	3.82 ± 1.17	2.78 ± 1.21	0.142
eGFR (ml/min/1.73m^2^)	13.51 ± 7.9	23.51 ± 21.21	0.036
Calcium (mg/dl)	9.62 ± 1.26	9.34 ± 0.79	0.382
Phosphorus (mg/dl)	6.13 ± 1.49	6.55 ± 1.13	0.354
PTH^*^ (pg/ml)	193.49 ± 101.25	144.79 ± 88.78	0.168
	(99/373)	(64/390)	
uOC-base (µmol/L)	499.86 ± 64.86	421.87 ± 120.45	0.047
uOC (µmol/L)	707.64 ± 111	475.31 ± 65.77	< 0.001
25 (OH) D_3_ (ng/ml)	15.36 ± 2.4	20.98 ± 6.46	0.002
BAP-base (IU/L)	244.71 ± 85.02	319.15 ± 124.49	0.062
BAP (IU/L)	438.71 ± 111.62	315.09 ± 134.17	0.019

^*^Data presented with interquartile range, Mann–Whitney test was used for comparison

*BAP* bone alkaline phosphatase; *BMI* body mass index; *BUN* blood urea nitrogen; *eGFR* estimated glomerular filtration rate; *PTH* parathormone; *uOC uncarboxylated osteocalcin*

In patients with fractures, serum uncarboxylated osteocalcin and bone-specific alkaline phosphatase were significantly elevated at the time of fracture compared to their baseline levels (*p* = 0.008 and 0.003, respectively).

In patients without fractures, bone-specific alkaline phosphatase was negatively correlated with 25(OH)D_3_ and PTH (*p* = 0.048 and p < 0.001, respectively) and positively correlated with P (*p* = 0.041). Conversely, in patients with fractures, bone-specific alkaline phosphatase showed a negative correlation with 25(OH)D_3_ (*p* = 0.015) but did not correlate with either P or PTH (*p* = 0.072 and 0.15, respectively) (Supplementary Fig. 2).

In patients with bone fractures, we observed a positive correlation between uncarboxylated osteocalcin and bone-specific alkaline phosphatase (*p* = 0.043), and a negative correlation with 25(OH)D_3_ (*p* = 0.017). Among patients without fractures, uncarboxylated osteocalcin showed negative correlations with both eGFR and 25(OH)D3 (*p* < 0.001 for both), while positive correlations were noted with P, bone-specific alkaline phosphatase, and PTH (*p* = 0.029, < 0.001, and 0.015, respectively) (Supplementary Table 1).

Logistic regression analysis was performed to ascertain the effects of uncarboxylated osteocalcin, serum Ca, P, bone-specific alkaline phosphatase, PTH and 25 (OH) D_3_ on the likelihood of bone fractures. Patients with a history of fractures were more likely to have higher uncarboxylated osteocalcin (*p* = 0.027, OR = 1.011). Serum Ca (*p* = 0.534), P (*p* = 0.250), 25 (OH) D_3_ (*p* = 0.230), bone-specific alkaline phosphatase (*p* = 0.437) and PTH (*p* = 0.223) were not significant.

**Fig. 2:**
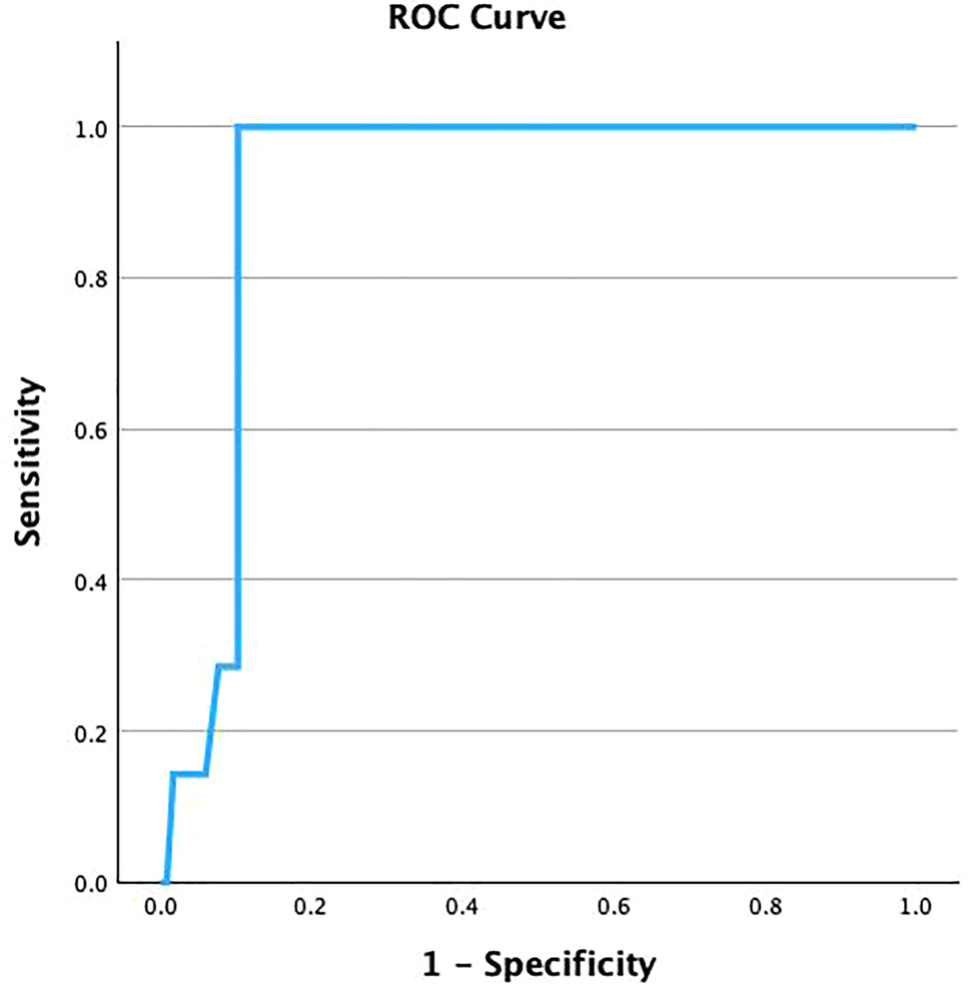
Receiver operating characteristic (ROC) curve of uncarboxylated carboxylation in the studied groups

By using ROC curve analysis, the diagnostic cutoff value for uncarboxylated osteocalcin was 628.5 (µmol/L) with accuracy 0.915, sensitivity 100%, and specificity 89.7%. The positive and negative predicted values of uncarboxylated osteocalcin were 0.38 and 0.88, respectively (Fig. 2).

## Discussion

Osteocalcin is a primary non-collagenous protein that contains 49 amino acids in humans and is synthesized by osteoblasts and hypertrophic chondrocytes [11]. When bone is resorbed by osteoclasts, the acidic environment in the resorption lacuna removes the carboxyl groups from osteocalcin, leading to the release of uncarboxylated osteocalcin into the bloodstream. As a result, the levels of circulating uncarboxylated osteocalcin are influenced by the rate of bone turnover (remodeling) [12].

Numerous prior studies, encompassing both adults and children, have established a relationship between biochemical indicators of vitamin K2 status and bone health [8, 13, 14]. In our study, we observed significantly elevated levels of uncarboxylated osteocalcin in the small number of patients with bone fractures. Moreover, uncarboxylated osteocalcin was the only predictor of bone fracture in this small group of patients. A study conducted by Kohmeier et al. [15] yielded similar findings. Their research delved into the function of uncarboxylated osteocalcin in bone metabolism, explored the correlation between uncarboxylated osteocalcin and vitamin K2, investigated the history of previous bone fractures, and assessed the risk of bone fractures in patients with kidney failure. Heightened bone turnover and reduced intake of vitamin K2 likely influence the bone's integrity, rendering it more susceptible to fractures [16]. This is reinforced by two observations in our study: firstly, the positive correlations between uncarboxylated osteocalcin and bone-specific alkaline phosphatase levels. Secondly, elevated uncarboxylated osteocalcin was found to be linked with a higher probability of experiencing bone fractures. These patterns were particularly evident among the CKD patients on regular HD in comparison to the CKD groups without need for KRT. The precise reason for these observations in the HD group requires further elucidation. However, it could be explained by reduced renal clearance and increased bone turnover as evidenced by high bone-specific alkaline phosphatase levels in those patients.

In a previous study, Nagata et al. observed a high uncarboxylated osteocalcin/intact osteocalcin ratio in HD patients. They explained this observation as being linked to a heightened release of osteocalcin molecules from the bone into the bloodstream. This was supported by the finding of a distinct connection between initial levels of serum bone markers and the rise in the serum uncarboxylated osteocalcin/intact osteocalcin ratio in HD patients during the inter-dialytic period [17].

One hypothesis that may partially explain the role of uncarboxylated osteocalcin suggests that elevated levels of uncarboxylated osteocalcin indicate accelerated bone turnover, leading to bone loss and reduced bone mineral density. This reduction in bone mineral density may subsequently result in osteopenia, increasing the risk of bone fractures [18]. In adults, it has been found that elevated levels of uncarboxylated osteocalcin are linked to a higher risk of osteopenia or osteoporosis in both men and women. Additionally, higher levels of uncarboxylated osteocalcin have been identified as a predictor of hip fractures in older women [19]. We may therefore suggest that uncarboxylated osteocalcin is a good predictor of bone fracture in CKD children, with high accuracy and sensitivity.

Bone-specific alkaline phosphatase is a sensitive marker of bone health, providing an indication of overall bone turnover. As expected, bone-specific alkaline phosphatase levels tend to be lower in low-turnover bone diseases and higher in high-turnover bone diseases, and correlate with PTH [20]. In our study, we observed a positive correlation between uncarboxylated osteocalcin and bone-specific alkaline phosphatase in patients both with and without fractures. We found a positive correlation with PTH in patients without fractures. However, we did not observe this correlation in the smaller group of patients who had fractures.

The two osteocalcin isoforms (uncarboxylated and gamma-carboxyglutamic osteocalcin) not only influence intracellular signaling but also impact the expression of parathyroid-specific genes. Cells derived from passaged adipose-derived stem cells responded to both gamma-carboxyglutamic osteocalcin and uncarboxylated osteocalcin stimulation by upregulating the expression of the parathyroid-specific gene PTH [21]. Our results support these findings, as we observed a positive correlation between uncarboxyated osteocalcin and PTH. This correlation was evident only in patients without fractures and not in those with fractures, which we believe is due to the small size of the fracture group.

In our research, we found a negative correlation between uncarboxylated osteocalcin and 25(OH)D_3_ in both fracture and non-fracture groups. The relationship between vitamin D and osteocalcin has gained interest among researchers. For example, Buranasinsup and Bunyaratavej reported a weak positive correlation between uncarboxylated osteocalcin and 25(OH)D_3_ [22]. Conversely, other studies, such as those by Szulc et al. [23] and Saadi et al. [24], found a negative correlation between uncarboxylated osteocalcin and vitamin D. Vitamin D directly stimulates osteocalcin transcription, while vitamin K regulates its carboxylation. The inconsistency in these findings may be due to the fact that levels of uncarboxylated osteocalcin are influenced by vitamin K status, whereas total circulating osteocalcin concentrations are affected by bone cell activity, independent of vitamin K [25].

This study supports uncarboxylated osteocalcin as a sensitive indicator of bone fractures in children with CKD. However, it is important to consider some limitations in our findings. First of all, the small size of the group of patients with fractures. Additionally, we used bone-specific alkaline phosphatase as a marker of bone health, though it has its limitations, with bone histochemical analysis and bone mineral density being the gold standard for assessing bone health.

Future research should investigate the therapeutic effects of combining vitamin K2 and vitamin D in CKD patients and especially in children.

## Supplementary Information

Below is the link to the electronic supplementary material.

Supplementary file1 (JPEG 26 kb)

Supplementary file2 (PNG 280 kb)

Supplementary file3 (DOCX 13 kb)

## Data Availability

All data generated or analyzed during this study are included in this published article and its supplementary information files.
