# Supplementary figures and images for: Osteocalcin as a predictor of bone fracture in children with chronic kidney diseases
Source: J Nephrol. 2025 Sep 3;38(9):2961–8. doi: 10.1007/s40620-025-02385-4 (PMC12712007; doi:10.1007/s40620-025-02385-4)

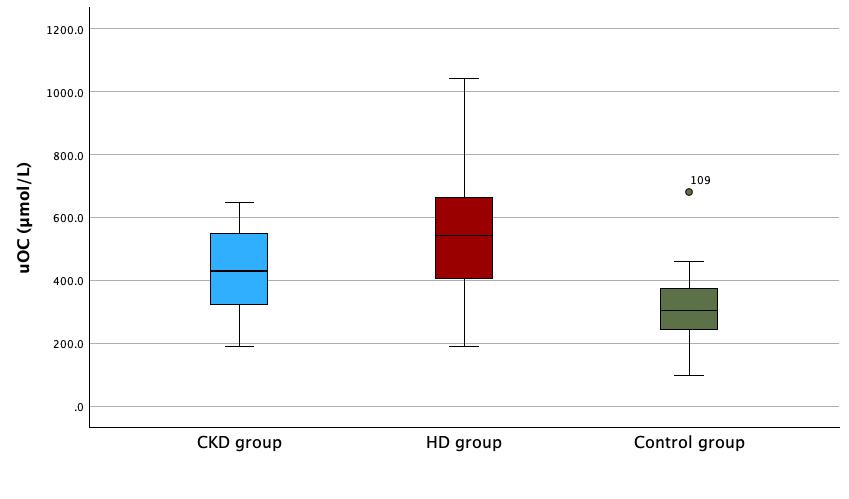

Supplement: Supplementary file 1 — Supplementary file1 (JPEG 26 kb) [file 40620_2025_2385_MOESM1_ESM.jpeg]

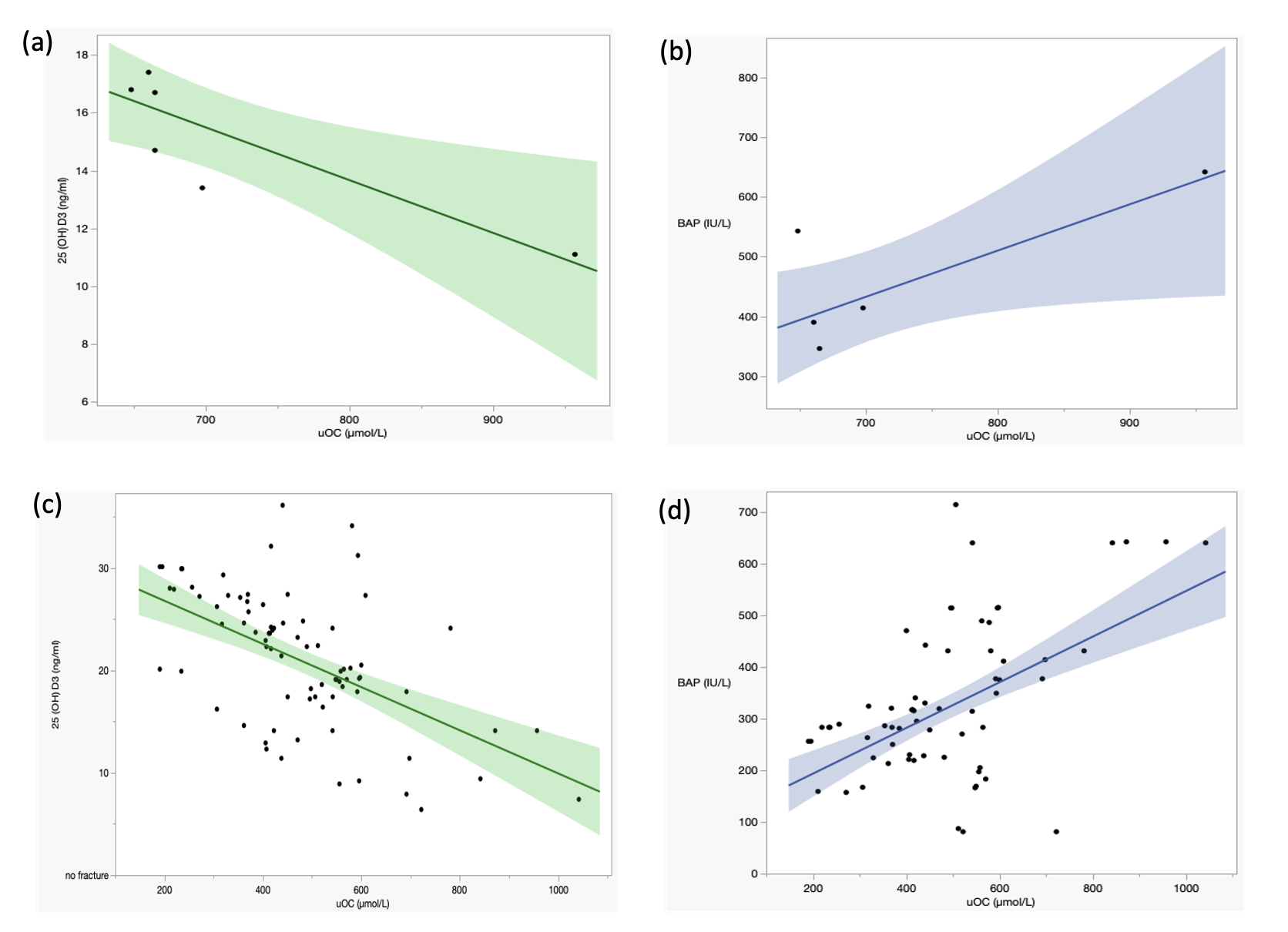

Supplement: Supplementary file 2 — Supplementary file2 (PNG 280 kb) [file 40620_2025_2385_MOESM2_ESM.png]
