# Supplementary material for: Osteocalcin as a predictor of bone fracture in children with chronic kidney diseases
Source: J Nephrol. 2025 Sep 3;38(9):2961–8. doi: 10.1007/s40620-025-02385-4 (PMC12712007; doi:10.1007/s40620-025-02385-4)
Supplement: Supplementary file 3 — Supplementary file3 (DOCX 13 kb) [file 40620_2025_2385_MOESM3_ESM.docx]

Supplementary Table (1): Correlation of uOC with different parameters in patients with and without fractures

|  | Patients with fractures  (N=7) | Patients without fractures  (N=95) |
| --- | --- | --- |
| eGFR  Calcium  Phosphorus  BAP  PTH  25 (OH) D3  BMI (z-score) | *p* =0.330  *r*= -0.434  *p* =0.888  *r*= -0.066  *p* =0.163  *r*= 0.590  *p* =0.043  *r*= 0.771  *p* =0.568  *r*= 0.263  *p* =0.017  *r*=-0.844  *p* =0.896  *r*= 0.062 | *p* <0.001  *r*= -0.540  *p* =0.460  *r*= -0.077  *p* =0.029  *r*= 0.228  *p* <0.001  *r*= 0.546  *p* =0.015  *r*= 0.248  *p* <0.001  *r*=-0.540  *p* =0.617  *r*= 0.052 |

BAP= bone alkaline phosphatase; BMI = body mass index; eGFR= estimated glomerular filtration rate; PTH = parathormone
